# Support and Empowerment for Older Adult Spousal Caregiving of People with Mild and Moderate Dementia: A Participatory Action Research

**DOI:** 10.3390/healthcare10030569

**Published:** 2022-03-18

**Authors:** Chia-Jung Hsieh, Pei-Fang Yin, Chi-Yi Chiu, Yu-Ping Hsiao, Yu-Ling Hsiao

**Affiliations:** 1 School of Nursing, College of Nursing, National Taipei University of Nursing and Health Sciences, Taipei 112303, Taiwan; 2 Department of Long-Term Care, Camillian Saint Mary’s Hospital, Yilan 265502, Taiwan; peifangyin@gmail.com; 3 Lezhi Home-Based Long-Term Care Institution, New Taipei City 220043, Taiwan; jelly520@hotmail.com; 4 Yue Xin Day-Care Center for Dementia and Child Development, Assessment and Intervention Center, New Taipei City 242033, Taiwan; hsiaopin32@gmail.com; 5 Center of Geriatric Care Resource, Fu Jen Catholic University, New Taipei City 242062, Taiwan; 125602@mail.fju.edu.tw; 6 Department of Nursing, College of Medicine, Fu Jen Catholic University, New Taipei City 242062, Taiwan

**Keywords:** empowerment, older adult, spousal, caregiver, mild and moderate, dementia, participatory action research

## Abstract

Background: Little attention has been given to the older adult caregivers of spouses with mild and moderate dementia in the caring dynamics process. The aim of this action research was to develop a program for providing support and empowerment to older adult caregivers of spouses with mild and moderate dementia in the community. Methods: The researchers acted as facilitators, with a view to empowering participants. We recruited participants from a day-care center and two community service stations. Data were collected with semi-structured, in-depth interviews with 19 dementia care dyads and from the notes, reflections, and feedback of collaborative researchers. Relevant themes for content analysis were extracted. Results: Three action cycles were completed over 18 months. The results revealed goals of three cycles: to connect the home situation and effective dialogue as a bridge to the researcher, to confirm the daily needs or expectations of the caregiver and the patient, and to enhance the interactions and quality of life of family members with resources and network. This process was cyclical and repetitive, and it also generated partnerships that built relationships among the interdisciplinary team, families, and researchers. At the same time, team workers formed a cooperative and coordinated family service mechanism to reflect the professional values and practice capabilities. Conclusions: The intervention program was based on the promotion of factors for the caregiver, linking to environmental protective factors, and the stabilization of mental and neurological symptoms of dementia patients, thereby enhancing the response capabilities of home caregivers while meeting the patient’s care needs in life. It is a tool that can effectively be used for support and empowerment in this population.

## 1. Introduction

Alzheimer’s disease and other forms of dementia rank as the seventh leading cause of death globally, and roughly five percent of the older population globally, approximately 50 million people, are estimated to have dementia [1]. East Asia is the region with the most people living with dementia (9.8 m) [2]. Taiwan is one of the fastest ageing countries in East Asia, and the high ageing rate results in increasing problems for the care of older adult members of society. In 2020, a survey from the Ministry of Health and Welfare (MOHW) revealed that Taiwan had 291,961 dementia patients aged over 65, and that the number is expected to increase at a rate of one new case every 48 h [3]. According to an annual report from Alzheimer’s Disease International (ADI), the vast majority of people with dementia live at home and wish to remain there, and approximately half of these have family members and caregivers living with them [4]. Over 70% of dementia patients are taken care of by a single caregiver or by their spouse [5]. The diagnosis and treatment of dementia not only result in worsened psychological states, relationship satisfaction, and health-related quality of life (HRQoL) in patients with dementia [6,7,8] but also affect their family caregivers [9]. The situation is more serious and challenging for aged caregivers of older adults with dementia [10,11].

Taiwan has become an aged society, for people over 65 years old now accounting for more than 16.15% of the country’s total population [12], and this group includes a large number of patients with dementia [13]. In aged societies with low birthrates, which are most countries worldwide, the potential support ratio has dropped [3,4,13]. Because family caregivers are expected to be the primary source of care going forward [14], there has been an increase in the number of older people who have become caregivers for their older adult relatives in the community [11]. In recent years, the number of older people who have become caregivers for their older adult relatives with dementia has increased [15].

Older caregivers of older patients with dementia experience a higher care burden when the patients have more greatly impaired functional autonomy and the Neuropsychiatric Inventory (NPI) symptoms of apathy and irritability, and most of these caregivers are the wives of the patients [10]. Spousal caregivers may sense a lack of support from their spouses with dementia [11,15]. A thematic analysis revealed various processes of reacting to a diagnosis of dementia: acknowledging change, being in crisis, adapting and adjusting, accepting, and moving forward in the spouses’ experiences of living with a partner with a dementia [16]. However, despite the variety of services and program models targeting the needs of individuals living with Alzheimer’s disease or other types of dementia and their spouses [17,18,19], fewer studies have been conducted on relatives of people with a recent diagnosis of mild to moderate dementia, so the effects of interventions on their partners remain unclear. In addition, little attention has been given to support and empower older adult caregivers of spouses with mild and moderate dementia in the caring dynamics process.

Therefore, this research engaged with people who live with dementia and with their spouses in the community to generate knowledge of the support they need to maintain their abilities and continue to live relatively normal lives for as long as possible. This study recognized people with dementia as experts on their own experiences. Rather than only fulfilling the role of providing health services in the community, the lead researcher wanted to enable people with dementia to break new ground by contributing to the creation of knowledge. Similarly, spouses who support people with dementia have an important contribution to make [20]. The aim of this action research was conducted to develop a program for support and empowerment among older adult caregivers of spouses with mild and moderate dementia in the community. The hypothesis of this project was that the challenges, demands, and obstacles for the older adult spousal caregivers of in-home patients with mild or moderate dementia could be resolved or improved after an action plan for caregiver empowerment intervention was developed and implemented. For the participating families, the problems and unexpected challenges that occurred during the process of understanding the effects of the empowerment intervention could be resolved.

This paper reported on a participatory action research study which arose out of the initiatives of people caring for partners attending dementia programs at one day-care center and two dementia community service stations. Through a democratic decision-making process, the day-care staff and family caregivers consulted with clients to design, implement, and evaluate a new therapy program. The researchers acted as facilitators in this process, with a view to empowering participants at each stage in the action research cycles. In the following sections, the professional and methodological approach to the research are described, followed by an outline of how actions pertinent to the research outcomes were informed by the philosophy of critical hermeneutics.

## 2. Materials and Methods

This project adopted an action research methodology to carry out the goal of “reflection” and “participation” at specific natural settings (the homes of patients with dementia). The action research approach is practically orientated with an emphasis on the integration of research theories with practical work. It mainly involves developing knowledge-based actions, verifying knowledge through action, or forming new knowledge in action. Through continuous attempts, reflection, and correction, the cycle of planning, acting, observing, reflecting, and other steps [21], a family empowerment-based intervention program suitable for patients with dementia was devised. This program was developed for providing support and empowerment to the older adult caregivers of spouses with dementia. In this program, the investigators served as facilitators and worked with the study participants (older adult patients with dementia and their caregivers), the directors of the Dementia Common Care Center, case managers, and other family members to jointly define the situation and problems encountered in the care process and to improve caregiving capabilities. Through the implementation of an action plan, the team pooled wisdom and methods to find solutions to improve the quality of care for current older adult families with dementia patients.

For the long-term care services, inter-professional collaboration is the cornerstone of successful implementation. In 2018, Taiwan started the dementia care policy, which emphasizes whole-person integrated care and attaches great importance to community-based care activities, the purposes of those activities were to enable the older people or the disabled to stay at home and to live in the community they know well, and to provide accessibility services through a network of formal and informal resources within the community [22]. Therefore, this intervention program was also linked with the government’s public sector policy external resources to form an inter-professional service. The inter-disciplinary team consisted of five members for all families, comprising a community nurse, a physiotherapist, a care manager, and two home care assistants. Our team members were trained and certified in dementia care.

During the research period, we also linked the services of the long-term care management information platform. When the case family needed the assistance of external professionals, our team also cooperated with geriatric physicians, social workers, psychologists, or pharmacists. This provided appropriate services for dementia cases and support for family caregivers.

### 2.1. Study Design

In this study, an action research framework was developed after consulting relevant literature regarding action research processes and practices [21]. The first cycle involved reflection on the initial actions, first placing attention on the home care situation to find care problems, the unmet demand, and obstacles. The home intervention action research program, empowerment strategies, and methods were then developed. At the same time, cooperative community service resources and recommendations on feasible programs were sought. Actions were then taken to implement the plan, monitor and collect information and evidence, and subsequently evaluate its effectiveness and incorporate corrective feedback. At this point, the second cycle started, which provided a reflection after the initial action. The family empowerment action research program continued to be altered based on revisions to care practices and focus areas. Once again, cooperative community resources were sought and suggestions were solicited. The action strategy was revised for the second time and put into practice. Appropriate data and evidence were collected through monitoring before the next cycle of evaluation and feedback was started. All cycles in this research were carried out at in-home care settings.

### 2.2. Study Setting

In this study, the setting of data collection took place at a dementia common care center and two dementia community service locations in northern of Taiwan. Based on the inclusion criteria, patients with dementia and their families currently living in the community were solicited to participate in this study.

### 2.3. Participants Recruitment

Based on purposive sampling principles and semi-structured interview guidelines, in-depth interviews with 19 pairs of participants (dementia patients and older adult.

Spousal caregivers) were conducted and recorded. The qualitative analysis software NVIVO 8 was used to extract the relevant themes and further summarize and analyze the themes, sorting them into categories. The data collection period was from August 2019 through December 2020, a period of 1.5 years. In addition to the interview contents, the investigator observed and recorded the participants’ family interactions during home visits and kept notes (Table 1). Such information, together with the discussion minutes from meetings with co-investigators and feedback records, were transcribed into a complete verbatim manuscript before the information was extracted. The data analysis process involved systemic analysis, generalization, and decoding procedures to specifically demonstrate the outcome of the home care empowerment intervention [23].

In this study, the inclusion criteria for family caregiver participants were: (1) aged 65 or older; (2) the spouse of a patient, (3) a major care provider for a patient for more than one year, and (4) able to communicate in Mandarin or Taiwanese and agreed to participate in the study. The exclusion conditions were: (1) younger than 65 years old or who were (2) experiencing a major mental illness (such as schizophrenia). For patient participants, the inclusion criteria were (1) living at home, (2) diagnosed with dementia by a physician in accordance with the Diagnostic and Statistical Manual of Mental Disorders (DSM-5) or the National Institute of Neurological and Communicative Disorders and Stroke and the Alzheimer’s Disease and Related Disorders Association (NINCDS/ADRDA), and (3) had used community care-related resources for more than half a year. The exclusion conditions were: (1) those who were confused or had delirium or (2) were staying at long-term care institutions or nursing homes.

### 2.4. Data Processing and Analysis

This study administered qualitative interviews and adopted the content analysis method [24]. The interviews and conversations centered around the care process and care needs of older adult patients with mild to moderate dementia. The interviews had a duration of 60 to 90 min. The first interview was an in-depth interview to collect demographic information. The interview was conducted using a content outline as a guide. A minimum of one in-depth interview with each pair of participants was conducted, supplemented with a 2nd or 3rd interview scheduled on a different day if the data collected were insufficient. However, depending on the situation, the investigator could stop collecting data when data saturation was reached, that is, when no additional new categories of information were being identified [24].

The outline of the semi-structured caregiver interview included the following elements: (1) Please introduce yourself; (2) How did the patient (he/she) begin to show symptoms of dementia? What was the dominant symptom they experienced, and how did you feel when taking care of that? (3) How long have you been caring for your spouse because of the dementia? What changes have you experienced in your life since you started to providing care? What difficulties have you had? (4) What has been the impact of caring for your sick spouse on your life? (5) How do you feel in the process of caring for your spouse? (6) What does this new life relationship (symbiotic care relationship) mean in each other’s lives? What differences do you find compared to the past? (7) In the current care process, do you need any help? Or what kind of care needs would you want to have fulfilled?

At the end of the research project, the findings, observations, and problem-solving outcomes of the program intervention implementation were shared. In other words, the investigators, family members interviewed, and research team members would exchange the results to acknowledge the growing awareness and actions of every family member and team member in empowering dementia care.

The semi-structured caregiver interviews were recorded and subsequently transcribed verbatim in Microsoft Word and software NVIVO 8. Using content analysis, the data were coded and categorized [24]. The data were read through several times and simultaneously the meaning units were tabulated for situations and solutions according to the semi-structured interview questions. Next, the meaning units were extracted as open codes to a table and searched for differences and similarities and categorized according to whom the situation of the challenges, demands, and obstacles occurred. The similar codes were combined and formed into subcategories and grouped by similar situations into a common upper category.

Regarding the trustworthiness of qualitative content analysis, the following are presented such as credibility, dependability, conformability, transferability, and authenticity [25]. To apply trustworthiness in the whole study, the researcher attempted to collect and analyze appropriate data with the maximum variety according to Lincoln and Guba criteria to reach saturation [26]. The process of data analysis was a reciprocal continuous rotational comparison, and to control any ambiguity, the data were controlled by the research members (Member Check). The codes obtained from the peer check and external check methods by the authors’ team were revised and modified in the presence of three experienced professors in the field of qualitative studies and dementia care experts.

## 3. Results

### 3.1. Participants

In this study, the spousal caregivers were in the age ranging from 66 to 78 years, while the patients, 13 with mild dementia and the others with moderate symptoms, were in the age ranging from 71 to 87 years. Six dementia patients regularly participated in daytime activities at the dementia common care center, while others took part in activities at the community dementia service locations irregularly, mainly with the assistance of their spouses in daily activities (Table 2).

### 3.2. The Plan Process of Goals of Action Research

**Figure 1:**
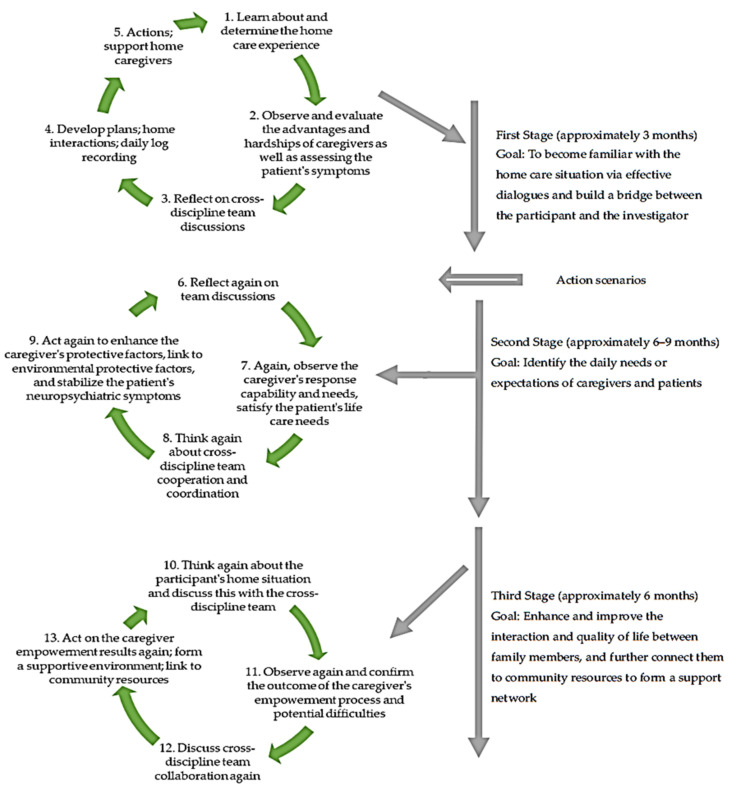
The planning process and goals of action research.

The process of action research can be divided into three cycles (Figure 1).

#### 3.2.1. The First Cycle

The goal of this stage was to become familiar with the home situation via effective dialogue and build a bridge between the participants and the investigators.

By establishing an initial relationship, this stage focused on the home life interactions between spouses to evaluate and understand the feelings and difficulties of the spousal caregiver through dialogue to find more information about (1) the mutual influence and symbiotic relationship in a family of two older adults; (2) responsibilities in providing care till the end of life; (3) problems derived from physiological changes with aging, empathy, lowered expectations, coping strategies in breaking through current hardships, and decisions in seeking medical help in the process (Top panel; Figure 1).

#### 3.2.2. The Second Cycle

The goal of this stage was to confirm the daily needs or expectations of caregivers and patients.

After extracting information from caregivers’ conversation transcripts, it was found that as the caregivers were busy with patient-related daily chores, he or she had the following care needs: (1) emotional support, (2) someone who could substitute or rotate in when the caregiver was physically exhausted and in need of rest, (3) enough rest at night, and (4) when in a healthy and functional mental state, the caregiver would like to have more knowledge about dementia care in order to continue playing the caregiver role to support the patient and further ensure the patient’s needs were satisfied. The caregiver also realized that when he or she was frustrated, his or her emotional distress may aggravate the patient’s symptoms because the caregiver and the patient influenced each other in their daily lives and were in a symbiotic relationship. Through communicating with the inter-disciplinary team, collaboration, and resource coordination at this stage, connecting the participants with appropriate resources can enhance family strength and promote the caregiver’s protective factors, coupled with environmental protective factors. By stabilizing the patient’s mental and neurological symptoms, the home caregiver’s response capability was strengthened while the patient’s care needs were met (Middle panel; Figure 1).

#### 3.2.3. The Third Cycle

This stage aimed to enhance or improve family interactions and quality of life and further connect the family with community resources to form a support network.

At this point, the previous support provided by the inter-disciplinary team was discussed, followed by additional observations of the home situation in order to identify the results or potential difficulties the caregiver encountered during the empowerment process. It was found that (1) more empathy and understanding of the patient’s illness can help the caregiver accept the current conditions, (2) expectations for the sick spouse were lowered, (3) excessive interference with the patient’s activities was reduced, (4) a balance between “self-needs” and “care responsibilities” was achieved, and (5) the expansion of the care support network and the acquisition of social resources made the caregiver feel more comfortable and meaningful in the care process. The quality of life in terms of day-to-day interactions was also improved (Lower panel, Figure 1). During the process, investigators and inter-disciplinary team members had opportunities to communicate while working on the same case and thus formed an intervention action team. In the current dementia care model, the results of empowering family caregivers were evident, and a collaborated and coordinated home service mechanism was formed, reflecting the action capabilities of different professional values.

### 3.3. The Action of Home Care Empowerment Program

**Figure 2:**
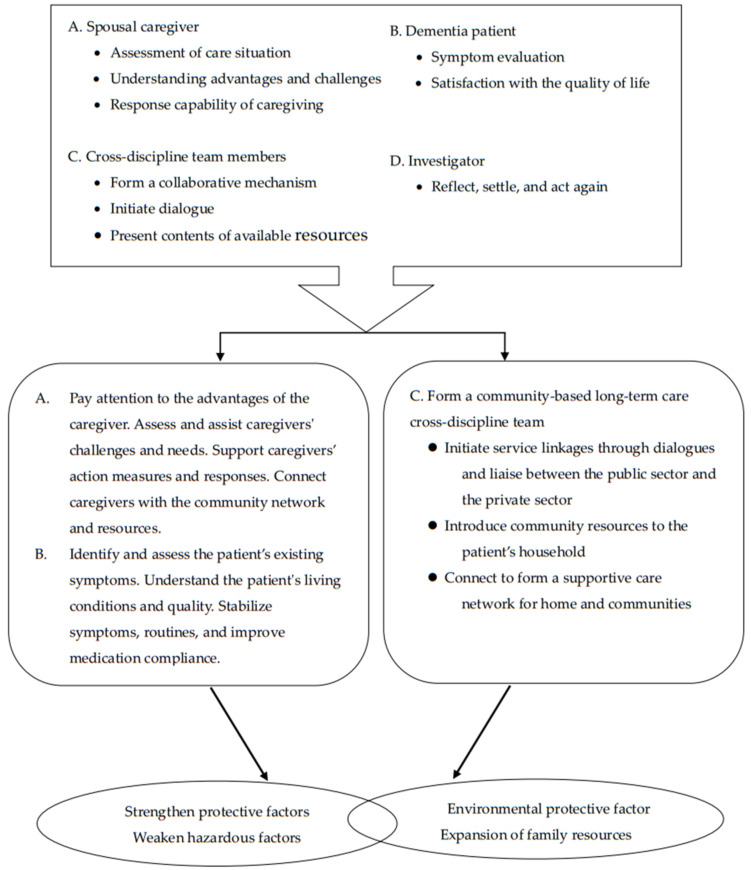
The action of home care empowerment program.

A participatory action research framework is embedded within the project design (Figure 2). The action research team meets monthly to review and reflect over activities and changes from the past months and plan for the forthcoming months. The reflections take the structure of discussing the month’s actions. Therefore, at the same time, team workers formed a cooperative and coordinated family service mechanism to reflect the professional values and practice capabilities. A summary of the home care empowerment program and the framework is depicted in Figure 2, showing the elements to support and empowering for spousal caregiver and dementia patient.

Figure 2 shows the protective factors that families with dementia patients need to strengthen. By assessing caregiver and patient needs and building on the family’s strengths, care-related problems can be solved to satisfy family needs. At the same time, it is necessary to reduce hazard factors, especially to maintain caregivers’ physical and mental health and improve medication compliance to stabilize the patient’s symptoms and daily routines; therefore, environmental protective factors can be formed and family resources can be expanded. Through public policies and communications with different organizations and groups in the private sectors, access to services can be expanded, and community resources can be introduced to caregivers to form a support care network for home and the community; thus, the family empowerment program can be implemented.

## 4. Discussion

This study is the first action research to explore the care process for older adult spouses with mild or moderate dementia and implement an empowerment intervention. The results of this study help to create an understanding of the care process and needs of older adult spouses with mild and moderate dementia. A family-based empowerment intervention program was developed. During the action research, the needs of family caregivers and patients were identified and the necessity to look at the advantages the family members had before the illness onset was recognized. The program provided caregivers with social support and resources so that the patient could continue to live in a familiar community.

In 2017, the WHO passed the Global Action Plan on the Public Health Response to Dementia, calling on governments of all countries to actively strengthen the care capacity for people with dementia, enhance family caregiver support networks, provide counseling and multiple supportive services, and increase the education and training of care skills to help relieve the burden of care [27]. In the first cycle, through the establishment of initial relationships, this study focused on the home life interaction experience between the spouses to evaluate and understand the feelings of the spousal caregivers and the difficulties experienced in life. The progressive symbiotic relationship between the spousal caregiver and the dementia patients observed in this study is consistent with previous studies [14,15]. The spousal caregivers’ caring process for dementia patients did not start at a given point in time, rather, it is a linear dynamic course originating from the period when his or her spouse began to experience symptoms of cognitive impairment. The spousal caregivers began taking care of their partners, who had increasingly impaired cognitive functions in their life together. The spousal caregiver started piecing medical treatments and diagnosis together and then undertook the care responsibility and mission [28]. The changes in life brought by caring for dementia patients affected the caregivers’ physical and mental health, well-being, and interpersonal relationships with others. The changes in the caregiver’s life also depended on his or her relationship with the dementia patient and the degree of participation in providing care [29,30]. In addition, among roles of different caregivers, such as a spouse, children, or other related persons, spousal care was the most troublesome [10]. Therefore, attention should be paid to the health issues of the spousal caregivers of dementia patients because they often were unable to practice health-promoting behaviors while facing their own physical changes that came with aging. Not only do they need to take care of the daily life of their spouse with dementia, but they also need to care for their own health needs. If professional medical assistance can be provided to them at this stage, it would not only help establish the relationship but would also enable the caregiver to find ways to break through the status quo and make better decisions and strategies.

In the home care of dementia patients, the existence of a symbiotic relationship in a family of two older adult individuals who mutually influence one another was found to be meaningful. Although the communication, intimacy, mutual benefits, and assistance in decision-making in a couple’s relationship greatly changed due to the disease [15,31], the spousal caregivers had a strong sense of responsibility for taking good care of their aging partners. This phenomenon may be attributed to Confucianism in Eastern society [17]. The spouse considered the caregiving task to be an inevitable responsibility. For patients with dementia, they were able to live continuously in a familiar home when they were supported by their spouses. A recent qualitative and integrated analysis pointed out that although spousal care has its advantages, “losing a partner” was still a strong feeling of the caregiver’s core emotions because the partner who suffers from dementia was no longer the same individual. Centered around this sentiment, spousal caregivers went through a disease acceptance process: acknowledging the changes in life, being in crisis, adapting, adjusting, accepting, and continuing to move forward [16]. During the process, professionals should be able to assist family members regarding how to maintain a new family balance, be keenly aware of the stages of personal growth as a result of the care process, and see the care task as a normal stage. Professionals should also provide family members with the medical resources needed to maintain good health. Another exploratory study of qualitative care trajectories pointed out that in the care process, spouses who took care of partners with dementia had a new perspective on their own lives in old age [32]. At the beginning of the care process, individuals may feel a big gap between themselves and the role of caregiver. Although their caregiving ability may not be very good, in the process of establishing the role, they found that they had become a person capable of taking care of their sick partner, and they had obtained self-care efficacy for their own life in old age [32].

In the second stage of the action research, the investigators developed an action plan based on the dialogue, observations, insights, and reflections of home care, which formed a basis for evaluating the effectiveness and responding to feedback [33]. In this process, the emotional needs of the spousal caregivers, expectations for care resources, the necessity to have a substitute, especially when the caregiver was exhausted, were discovered. Through conversation, it was evident that nightly sleep satisfaction was their basic need. A systematic review article on the sleep quality in caregivers for dementia patients pointed out that caregivers often did not get enough sleep, and the burden of caregiving deteriorated their sleep quality [34]. Insufficient rest at night may affect the quality of care during the day, and the caregiver may be prone to accidents. Therefore, more attention should be paid to the health status of older spousal caregivers to avoid the functional deterioration that may be caused by caring for disabled partners [35]. In the home care service field, professionals need to actively pay attention to the sleep quality of patients and older adult spousal caregivers. Through communications and collaborations with inter-disciplinary team members, the safety of the home environment should be assessed to provide an environment conducive to good sleep, thereby promoting the development of protective factors for caregivers linked to environmental protective factors. This health requirement can be effectively improved through sleep behavioral interventions.

On the other hand, the study showed that the neuropsychiatric symptoms of dementia patients brought many challenges and distress to the spousal caregivers in the care relationship, especially the negative emotions caused by not knowing how to deal with the behavioral changes related to dementia. Many family members in the community have major problems associated with patient care because they fail to actively allow dementia patients to receive treatment as they lack an understanding of the concepts of the disease and had insufficient experience in managing symptoms or stereotypes [36,37]. In addition, older adult caregivers may not receive information as easily as young caregivers; therefore, they tended to provide day-to-day patient care using their common sense [38]. Previous study has pointed out that when these caregivers gained more knowledge and understanding about dementia, they were more empathetic and closer to the patient, and their fear and negative emotions about symptoms were reduced, and they also had the knowledge and ability to care for patients, leading to a better quality of life [20]. A study on spousal caregivers pointed out that after implementing 6–12 months of cognitive-behavioral therapy and life review intervention, the caregivers were able to improve patient care efficacy [37]. In the intervention, the caregivers engaged in training activities with a focus on “finding meaning in everyday”, participants were able to correctly recognize dementia symptoms with the use of memory-improving aids and maintain social activities and contact with others in hope for a better future [37].

In the process of supporting the empowerment of home caregivers, it should be emphasized that caregivers did not passively accept professional suggestions but rather actively selected meaningful and executable information from recommendations and then took the appropriate actions [39]. In other words, in this study, the intervention strategy was participant-centered, emphasizing the philosophy of sharing instead of the philosophy of compliance. The idea of empowerment is an important concept in action research [33]. This study enhanced the ability of individual participants to set specific goals; promoted their capacity to adapt to an environment that cannot be changed; and assisted them in connecting with current community resources, searching for resources, and obtaining appropriate social support [39]. This study also took advantage of the mutual influence of family members to change the overall family function and health. With the growing awareness of decision-makers, assessment of family problems, resource connections, implementations, and actions, the caregiver’s inner strength and sense of control of the situation were improved, and the strengths of the family were increased [40]. This empowerment process not only strengthened the responsiveness of the home caregivers but also met the life care needs of the dementia patients.

The application of the empowerment concept began with the formation of partnerships between patients and caregivers, implying the establishment of mutual respect and trust. The health professionals had to encourage the participants to reflect and review care experiences, learn critical thinking, and make decisions that were conducive to health. Ultimately, through feedback sharing, the caregiver’s confidence in providing care for dementia patients increased and their self-efficacy improved. Consequently, the caregivers were able to stay motivated and maintain behaviors for managing their own health [39]. Therefore, in the third stage, the goal of the research team was to improve family interactions and quality of life, then connect the family to community resources to form a support network. In Taiwan’s “Long Term Care 2.0” strategic plan, the care of dementia patients is considered the highest priority. Since 2017, each county and city has set up a “Dementia Common Care Center”, which combines medical expertise and community care resources to assist the public in understanding the treatment and care for people with dementia, as well as providing greater support and services to family members [41]. At this stage, based on prior discussions with inter-disciplinary team members, the research team identified the difficulties the caregivers may encounter in the empowerment process.

According to the caregivers’ self-reflections, the symbiotic care relationship was meaningful to their lives. Many spousal caregivers mentioned that they lowered their expectations of sick partners, allowed them to have autonomy, and decreased excessive interferences with their behaviors. This not only helped caregivers accept the patients’ conditions, but also helped strike a balance between “self-demand” and “care responsibilities.” The study aligned with the WHO’s “Global Action Plan on the Public Health Response to Dementia” in which the empowerment and participation of dementia patients and caregivers, an important action of policy implementation, was mentioned [27].

During the research process, the investigators and inter-disciplinary team members established a team for the action interventions. This formed a collaborative and coordinated in-home service mechanism in the current dementia care mode, particularly for maintaining the mental function of the caregiver, improving the patient’s medication compliance, and stabilizing the patient’s symptoms and daily routines. By implementing the home care policy for patients with dementia, the program guided the spousal caregivers to communicate and connect with community organizations or workers and allowed community resources to be channeled into the patient’s home, thereby linking the family with the community support networks while allowing the patient to continue living in a familiar community [42]. Such an arrangement forms an important foundation for a dementia-friendly care environment [4]. The findings of this study are similar to the results of previous studies in that empowered intervention-based activities can improve caregivers’ self-esteem and perception of care work [43]. Thus, spousal caregivers can improve the health-related quality of life, increase self-efficacy, and improve their ability to solve everyday problems associated with the care of dementia patients [18].

In the process of this study, the investigators and team members liberated themselves from professional authority and autonomy, upheld the principles of equality and respect, and formed partnerships with the caseworkers in the field of home care. They recorded their deliberations in the research log, which provided insights, reflections, and an understanding of the problems related to the home care situation of patients with dementia. Such information can be used as a reference for the evaluation of the program’s effectiveness and modifications. This study also allows continuous and cyclic dialectics and adjustments of a three-stage empowerment intervention for home care of dementia patients, namely the research cycles of continual planning, acting, observing, and reflecting.

Furthermore, there is an issue worth pondering in this study, that is, the family view of Confucian philosophy. In eastern culture, harmony and integrity of the family is emphasized, and the care of the older adult should be given priority to family care [44]. However, as the social economy develops, adult children typically leave home to work after becoming independent, resulting in an increase in reliance on each other as the family’s older parents age. At this time, more external and human resources are needed to be able to be ageing in place, especially in the urban cities of northern Taiwan. The connection of external resources and the provision of professional assistance in older patients’ care are more important at this time. Therefore, when the older parents live in rural cities, due to the close trust between neighborhoods, there may be some differences in living needs and community resources, which may require further research.

### Strengths and Limitations

In this study, four elements that appear to be important in enhancing success emerged. The first is contextualization; a study must be adapted to the context wherein local community services and partners operate. Only when the family partners perceive the project as valuable can they be expected to engage extensively. Second, trust needs to be built during the collaboration process with the families, and respect and understanding among professional partners and family members must be created. Third, the accessibility of project activities for all families and partners must take language and culture into consideration. This also creates a certain feeling of empowerment and was found beneficial to engagement. Finally, an open mind and transparency are especially important for professionals, family partners, and researchers. We should communicate openly with the spousal caregivers and respond to their needs and priorities.

Although the results of this study provide useful information about older adult spousal caregiving in people with mild and moderate dementia and their interactions with an interdisciplinary team in the dynamic process, the following limitations should be considered. First, information regarding the status of the participants’ family members with dementia was limited. The content analysis method that was selected had a potential for bias regarding the researchers’ implications when they were analyzing the data and drawing conclusions. Further research is needed to design a study with analysis triangulation. Second, the disease progression of the dementia patents might have contributed to the participants’ subjective perceptions or experiences. Third, the time required to build relationships, collect anecdotes from family members, and understand their experiences does not fit neatly into this intervention. From a future long-term perspective, it would be interesting to contemplate the application of empowerment, as well as to examine the effectiveness of the intervention when applied differently. Fourth, local community context factors, such as family friendly policies and practices, may confound or change the connections between aged spouses’ experiences and their negative effects, for different urban agglomeration networks have different characteristics and structures.

## 5. Conclusions

The aim of this study was conducted to develop a program for support and empowerment among older adult caregivers of spouses with mild and moderate dementia in the community. This study is the first action research to implement an empowerment intervention and explore the care process of older adult spousal caregivers for patients with mild and moderate dementia. In this study, via continuous attempts, reflections, and modifications, the project team developed an intervention program based on family empowerment. The process includes a cycle of steps, including “planning”, “acting”, “observing”, and “reflecting.” The results of this study demonstrated three stages of objectives in a family care empowerment program. The intervention program was based on the promotion of factors for the caregiver, linking to environmental protective factors, and the stabilization of mental and neurological symptoms of dementia patients, thereby enhancing the response capabilities of home caregivers while meeting the patient’s care needs in life. The multifaceted outcomes and intervention effectiveness derived from this study can be used as a reference for the future development of home care policies for dementia.

## Figures and Tables

**Table 1:** Contents in the research log of the family interaction process.

Item	Content
Date	Record the dates to show the continuity of the care process
Day-to-day care issues	Let the participants express the care issues about getting along with the patient in their own words.
Data collection	List all objective and subjective data (from the patient), such as daily observations, doctor visit instructions, or related examination reports.
Assessment	Assess the patient’s physical, psychological, social, and mental state.
Community care management	List the activities or nursing guidance of the community care plan.
Implementation	Execute and describe the patient’s condition after the implementation.
Evaluation	Describe the patient’s post-care condition.

**Table 2:** Characteristics of caregivers of charges with Alzheimer’s disease.

	*n* (%)	Mean (SD)	Range
Caregivers’ age		73.16(3.95)	66–78
Gender			
Female	12(63.2)		
Male	7(36.8)		
Caregiving duration: months		19.84(4.26)	
Patients’ age		77.21(4.18)	71–87
Gender			
Female	7(36.8)		
Male	12(63.2)		
Dementia Level			
Mild	13(68.4)		
Moderate	6(31.6)		

Abbreviations: SD, standard deviation.

## Data Availability

This study represents ongoing research. Data are qualitative transcripts which contain details that could risk the anonymity of participants. They will not be made available.
